# Circall: fast and accurate methodology for discovery of circular RNAs from paired-end RNA-sequencing data

**DOI:** 10.1186/s12859-021-04418-8

**Published:** 2021-10-13

**Authors:** Dat Thanh Nguyen, Quang Thinh Trac, Thi-Hau Nguyen, Ha-Nam Nguyen, Nir Ohad, Yudi Pawitan, Trung Nghia Vu

**Affiliations:** 1 Department of Medical Epidemiology and Biostatistics, Karolinska Institutet, Stockholm, Sweden; 2 University of Engineering and Technology, Vietnam National University in Hanoi, Hanoi, Vietnam; 3 Information Technology Institute, Vietnam National University in Hanoi, Hanoi, Vietnam; 4 School of Plant Sciences and Food Security, Tel Aviv University, Tel Aviv, Israel

## Abstract

**Background:**

Circular RNA (circRNA) is an emerging class of RNA molecules attracting researchers due to its potential for serving as markers for diagnosis, prognosis, or therapeutic targets of cancer, cardiovascular, and autoimmune diseases. Current methods for detection of circRNA from RNA sequencing (RNA-seq) focus mostly on improving mapping quality of reads supporting the back-splicing junction (BSJ) of a circRNA to eliminate false positives (FPs). We show that mapping information alone often cannot predict if a BSJ-supporting read is derived from a true circRNA or not, thus increasing the rate of FP circRNAs.

**Results:**

We have developed Circall, a novel circRNA detection method from RNA-seq. Circall controls the FPs using a robust multidimensional local false discovery rate method based on the length and expression of circRNAs. It is computationally highly efficient by using a quasi-mapping algorithm for fast and accurate RNA read alignments. We applied Circall on two simulated datasets and three experimental datasets of human cell-lines. The results show that Circall achieves high sensitivity and precision in the simulated data. In the experimental datasets it performs well against current leading methods. Circall is also substantially faster than the other methods, particularly for large datasets.

**Conclusions:**

With those better performances in the detection of circRNAs and in computational time, Circall facilitates the analyses of circRNAs in large numbers of samples. Circall is implemented in C++ and R, and available for use at https://www.meb.ki.se/sites/biostatwiki/circall and https://github.com/datngu/Circall.

## Background

Circular RNA (circRNA) molecule identified in recent years is characterized by covalently closed-loop structures with neither a 5′ cap nor a 3′ poly (A) tail [1]. It is derived from the back-splicing of linear-transcript exons during the RNA-splicing processes [2, 3]. The circRNA has been gaining attention in cancer research recently since researchers discovered its potential role as an ‘miRNA sponge’ that suppresses the activities of oncogenic miRNAs such as miR-21 and miR-221 [4]. CircRNA is also found to be a regulatory factor during alternative splicing of pre-mRNA [5], and able to translate into proteins [6]. With its varied biological functions, circRNA is potentially useful as a biomarker or therapeutic target in future personalized medicine for human diseases [7].

Whole transcriptome sequencing by RNA sequencing (RNA-seq) technologies allows efficient discovery of circRNAs. It is mainly based on the detection of reads containing a back-splicing junction (BSJ), where the end of an exon joins to the start of itself or of another exon from the same gene. Several tools have been developed for detecting circRNAs using RNA-seq data. Comparative studies of these methods have been performed [8–10]. They usually have two main steps: (1) detection of circRNA candidates and (2) elimination of false positives (FPs). The first step employs standard mapping approaches such as STAR [11], Bowtie [12] and BWA [13] for read alignments. There are two main approaches to detect BSJ supporting-reads for circRNA candidates from RNA-seq data, including split-alignment-based and pseudo-reference-based strategies [14]. The former splits a read into small fragments to map against a reference genome. Then the reads having fragments mapped to exons with opposite orientations are identified as BSJ supporting-reads [15]. The latter directly maps RNA-seq reads to the prebuilt BSJ pseudo sequences which are constructed based on an assumed genome annotation [16, 17].

However, detected BSJs are not specific to genuine circRNAs. Transcripts with duplication of exons caused by exon repetition, genomic tandem duplication or technical artefacts including trans-splicing, and reverse-transcriptase (RT) template-switching (Additional file 1: Fig. S1), can also produce FP BSJs which have nothing to do with circRNAs [8–10, 18]. This issue will be also discussed further in the next section. For convenience, from now on these FP BSJs will be considered as tandem RNAs. To reduce FPs, in step 2, current detection methods apply various filters such as canonical-splicing signals, the minimum number of BSJ supporting-reads, unique anchor alignments, the limited distance between the two splice sites and paired-end read consistency [3]. Besides, some tools such as KNIFE and CIRI2 introduce statistical tests in the alignment process to avoid wrong alignments [19, 16].

Despite these efforts, there are still serious challenges in circRNA detection. Firstly, the existing tools often have little overlap in the results [18]. Secondly, these methods rely on the number of BSJ supporting-reads, which is not a reliable statistic for the detection of genuine circRNAs [18]. Finally, they still produce many false positives due to the tandem RNAs [8–10, 18].

To address these challenges, we have developed a novel tool called Circall for circRNA detection from RNA-seq data. It uses a robust statistical method based on the two-dimensional local false discovery rate [20]. It is computationally highly efficient because it employs quasi-mapping for fast and accurate RNA read alignments. We have applied Circall and compared it with several leading methods in the analyses of several datasets, including two simulated and three validated experimental datasets. The results from the simulation study indicate Circall’s high sensitivity and precision, while in the analyses of three experimental datasets of human cell lines, Circall performs well against the existing methods.

## Materials and methods

**Fig. 1:**
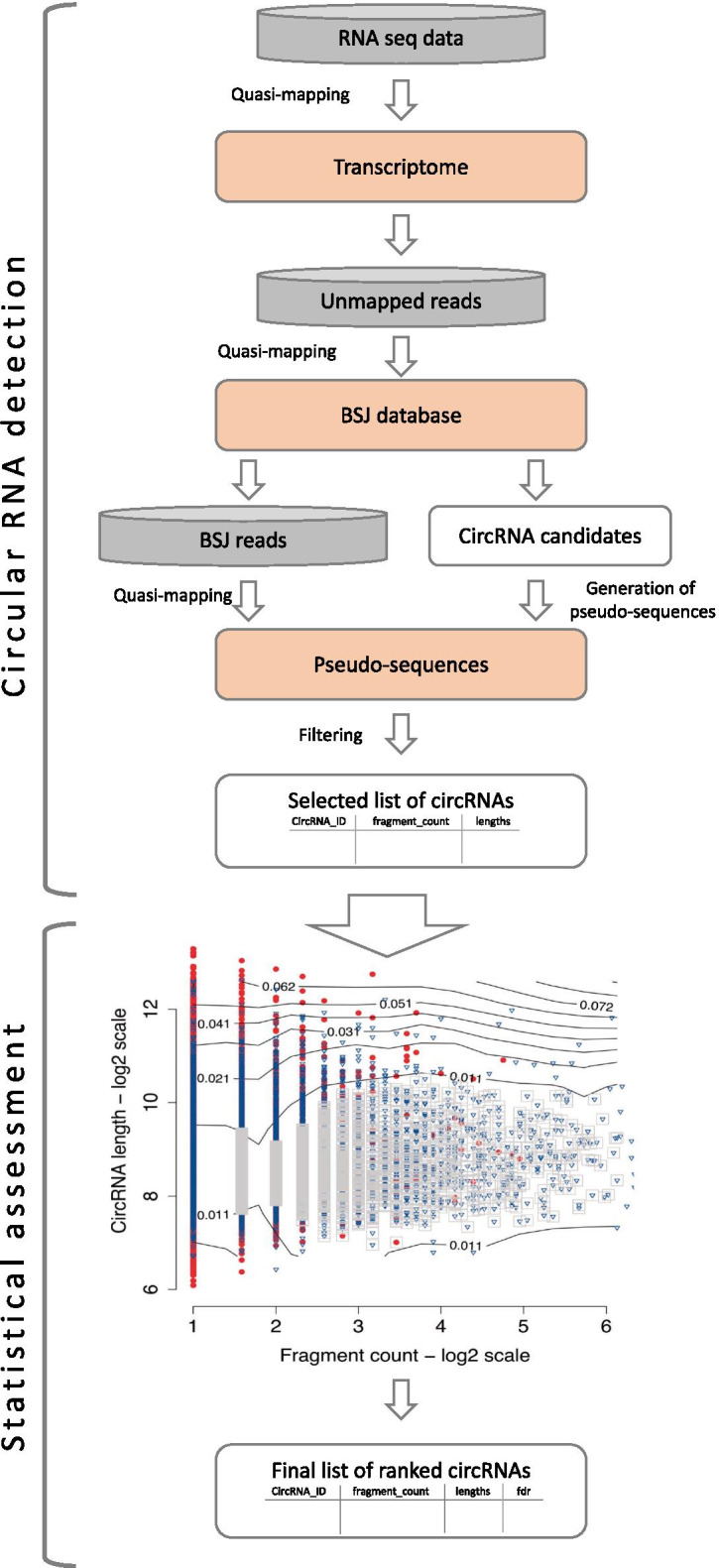
Overview of Circall for the discovery of circRNA from RNA-seq: (i) CircRNA candidate detection to discover a list of circRNA candidates. This includes extraction of unmapped reads, collection of BSJ reads, generation of pseudo-sequences of circRNAs and potential tandem RNAs, and filtering to get the list of candidates; (ii) Statistical assessment to rank the candidates. The contour map is an example from the Hela dataset for the statistics from the permutation in the 2d local false discovery rate (2dfdr) method. The red dots and blue triangles indicate the depleted and non-depleted circRNAs, respectively. The circRNAs with fdr2d are marked by grey squares. Details are described in the main text

The overview of Circall for the discovery of circRNA from RNA-seq is presented in Fig. 1. The method includes two key steps: (1) circRNA candidate detection and (2) statistical assessment. In the first step, all input reads are mapped to the annotated transcriptome to remove the reads from linear transcripts and to extract unmapped reads. Next, these unmapped reads are mapped to a BSJ reference database pre-built from the annotated transcriptome in order to find reads supporting BSJ and circRNA candidates. Finally, these BSJ-supporting reads are mapped to pseudo-sequences of circRNAs and potential tandem RNAs to exclude FP reads, and generate a list of circRNA candidates. These are then statistically assessed and ranked according to their two-dimensional local false discovery rates. Further details are presented next.

### CircRNA candidate detection

This step is used to discover circRNA candidates based on their BSJs. Circall detects circRNAs using a reference-based approach. In this approach, sequences of all BSJs of all potential exonic circRNAs are generated from gene annotation and used as a reference for read alignment. Circall uses the ultra fast quasi-mapping tool RapMap [21] to perform the read alignment.

#### Building BSJ reference database

To collect the BSJ supporting-reads, we build a database containing the BSJ sequences of all possible exonic circRNAs from the gene annotation. Specifically, for each gene, the start and end positions of all exons are identified (Additional file 1: Fig. S2A). Then, a matrix of all combinations of the start positions and end positions is constructed (Additional file 1: Fig. S2B). Only the combinations where the end position is greater than the start position are kept as potential BSJs. Next, the BSJ sequences of these combinations are generated together to construct the BSJ reference database. We make sure that BSJ supporting-reads can cover BSJ junctions. For example, to allow for RNA-seq protocols that have reads up to 150bp long, it is necessary to join 149 bases upstream of the end position to 149 bases downstream of the start position to generate the BSJ sequences (Additional file 1: Fig. S2C). Finally, the BSJs are filtered out using the canonical-splicing condition. Specifically, we keep only BSJs with GT-AG, GC-AG, and AT-AC junctions, which cover 99.24%, 0.69%, and 0.05% of the splicing events, respectively [22].

#### Detecting BSJ supporting-reads

To detect BSJ reads, the input RNA-seq paired-end reads are first mapped to the annotated transcriptome to separate out the wild-type reads and extract unmapped reads. The wild-type reads are the reads which are completely mapped to linear RNAs in the annotated transcriptome reference. Thus, they are likely not BSJ supporting-reads and should be excluded in the subsequent steps. Next, all single-end reads of these unmapped reads are mapped to the pre-built BSJ reference database. The mates of the BSJ supporting-reads and the mapping information are collected. Finally, the supporting reads are filtered by several conditions: at most 1% mismatch in a read is allowed (maximum 1 mismatch per 100 bases); the reads map uniquely to a single BSJ; and the minimum anchor length – the shorter piece of the read that straddles the junction is 10 bases.

#### Identifying FP BSJ reads from tandem RNAs

**Fig. 2:**
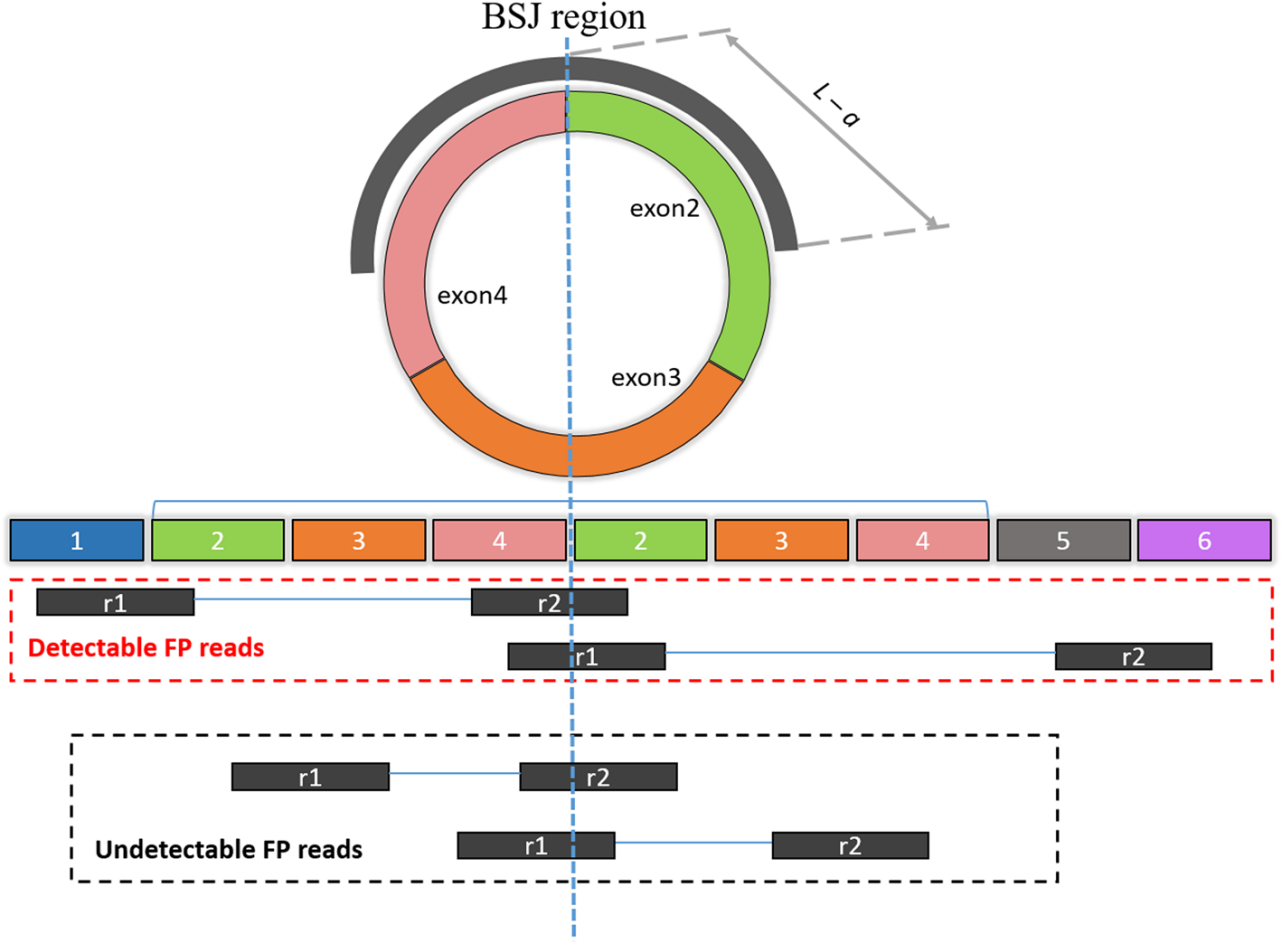
Identifying FP BSJ reads from tandem RNAs. Mapping location of the read mate of a BSJ-supporting read may help to detect FP BSJ reads. The vertical blue dash-line represents the location of BSJ in the true circRNA (top) and the tandem RNA (bottom). The BSJ region is surrounding the BSJ of the circRNA, defined by the area of ($L-a$) bases from the BSJ, where *L* and *a* are read length and anchor length respectively. All read pairs are originally from the tandem RNA. Upper dashed-box: BSJ read-pairs with one read mapped outside the circRNA putative region identified as false positives (FPs). Bottom dashed-box: read pairs from short fragments are completely located in the putative circRNA region, thus in this case the reads are not identified as FPs

To detect BSJ supporting-reads, input reads are mapped to the BSJ reference database as aforementioned in the previous step. However, BSJ supporting-reads are not guaranteed to come from genuine circRNAs. The aim of this step is to identify likely FP BSJ supporting-reads from tandem RNAs. The idea is illustrated in Fig. 2: The true circRNA is constructed by three exons: exon 2, 3 and 4, while the tandem RNA is constructed by 6 exons from 1 to 6, but the region from exons 2 to 4 is duplicated. All read pairs are originally from the tandem RNA. For the read pairs in the upper dashed-box, one read is mapped to the BSJ region, and the other read is mapped outside the circRNA region. This means the pair cannot come from the circRNA. In this case, the FP BSJ reads are detectable by computational approaches. For the paired reads in the lower dashed box, both reads are inside the circRNA region, so the mapping information alone cannot tell if the read-pair comes from a true circRNA or from a tandem RNA.

Circall identifies FP BSJs by mapping the BSJ reads to pseudo-sequences of both circRNAs and the corresponding tandem RNAs. Pseudo-sequence of a circRNA is generated by adding $L-1$ last bases of circRNA sequence to the beginning of the circRNA sequence (Additional file 1: Fig. S3), where *L* is the read length of the RNA-seq data. For circRNA candidates with more than 2 exons, the information of the alternative splicing from the linear transcripts from the annotated transcriptome is applied. Otherwise, the pseudo-sequences of circRNAs and tandem RNAs are collected from the sequences of all constituted exons. Only BSJ reads that have the corresponding read mates fully mapped to the putative circRNA region are kept in the list of candidate circRNAs. Some candidates may have BSJ supporting-reads that are both inside and outside a circRNA region. A recent study [23] shows that when the expression of circRNA is dominant, 20% to 35% of BSJs in humans arise from both circRNAs and trans-splicing RNAs. Therefore, we exclude candidates whose number of reads from its tandem RNAs is greater than 1/3 of the total number of BSJ supporting-reads.

### Two-dimensional local false discovery rate method

The expression level and length of circRNA are two key features that have been used in various ways to reduce FPs. For instance, many methods choose a minimum of 2 BSJ supporting-reads as a fundamental filtering criteria [3]. Also, in evaluation of circRNAs from several comparative studies [8–10], the number of BSJ supporting-reads is used to rank circRNA candidates. A recent study [10] also supports this idea by showing that, in all algorithms, *bona fide* circRNAs have higher expression than FP circRNAs.

Length is also a natural feature of circRNAs that can be used for filtering. Some studies [16, 18, 24–26] show that a spliced circular molecule can range from smaller than 100 to larger than 4000 nucleotides, but the most common circRNAs in human cells are a few hundred nucleotides long. Indeed, current methods such as Mapsplice [27], circRNA_finder [28] and CIRI [19, 29] indirectly utilise the length feature by setting the hard thresholds for the minimum and maximum distances between two circRNA splicing sites.

Estimating the exact length of a circRNA transcript is not straightforward due to alternative splicing events [30, 31]. We assume the structure of circRNA is generally based on the structure of a linear transcript. Thus, if there is more than one linear transcript supporting the BSJ of a circRNA, the length of the circRNA is computed by the median length of all possible circRNAs derived from those transcripts.

Circall controls the rate of false positives by utilising the information of both the number of BSJ supporting-reads and the corresponding circRNA length in the two-dimensional local false discovery rate (2dfdr) method. The methodology [20] was originally developed for microarray data analysis, but the concept can be applied in our current problem. The false discovery rate is a key statistical assessment for high-throughput data analysis that takes multiplicity into account. The local false discovery rate (fdr) is defined as

1 $$\begin{aligned} \text{fdr} (z) = \pi _0 \frac{f_0(z)}{f(z)}, \end{aligned}$$

where *z* is a statistic, $\pi _0$ is the proportion of the statistics that originate from the null hypothesis, $f_0(z)$ the probability density of the statistic under the null hypothesis, and *f*(*z*) the probability density of the observed statistic. The fdr is interpreted as the expected proportion of FPs among the discoveries that have observed statistics $Z \approx z$ [20, 32, 33].

For circRNA detection, we consider a 2-dimensional statistic $z=(z_1,z_2)$, where $z_1$ is the number of BSJ supporting-reads, and $z_2$ the length of the corresponding circRNA. Computationally, both read counts and circRNA lengths are transformed into log2 scale. The fdr2d based on $z=(z_1,z_2)$ of a circRNA candidate is defined as

2 $$\begin{aligned} \text{ fdr2d }(z_1,z_2)\equiv \pi _0\frac{f_0(z_1,z_2)}{f(z_1,z_2)}, \end{aligned}$$

where $f_0(z)$ is the 2d-density function of *z* from the null circRNAs, and *f*(*z*) the marginal density from all candidate circRNAs. The parameter $\pi _0$ is the proportion of null circRNAs. For convenience, we set $\pi _0=1$, which is conservative since that is its maximum value.

Since the fdr2d is the proportion of null circRNAs at the observed statistic *z*, it measures the rate of false discoveries if we consider the circRNA candidates with observed $z=(z_1,z_2)$ as true circRNAs. In practice we consider the depleted circRNAs as the null circRNAs, and the non-depleted circRNAs as the true positive circRNAs. Specifically, circRNAs are classified as non-depleted if their expressions in an RNase R– sample are less than or equal to those in the corresponding RNase R+ sample, otherwise they are assigned as depleted circRNAs.

Computing fdr2d requires estimates of $f_0(z)$ and *f*(*z*). The latter is readily available by non-parametric smoothing of *z*’s from the candidate circRNAs. Suppose there are *n* candidates with the corresponding *z* values, and let ${\mathbf{Z}}$ be the vector of *n* observed *z* values. To evaluate $f_0(z)$, we generate *z*’s by Monte Carlo sampling from known depleted/null circRNAs in other independent datasets. Specifically, we take *M* random samples, each of size *n*, and denote these samples as ${\mathbf{Z}}_1^*, \ldots ,{\mathbf{Z}}_M^*$. These represent samples of ${\mathbf{Z}}$ under the null hypothesis. In all experiments, we use $M=100$ samples.

Instead of estimating the densities separately, we found that the ratio estimation is more stable if we use the following procedure: first the ratio

$$\begin{aligned} r(z) \equiv \frac{Mf_0(z)}{f(z)+Mf_0(z)}, \end{aligned}$$

then computes the fdr2d as

3 $$\begin{aligned} \text{ fdr2d }(z) = \pi _0 \frac{r(z)}{M\{1-r(z)\}}, \end{aligned}$$

In order to estimate the 2d-estimation of *r*(*z*), the procedure

1. Considers all the statistics from ${\mathbf{Z}}_1^*, \ldots ,{\mathbf{Z}}_M^*$ as ‘successes’ and the observed statistics from ${\mathbf{Z}}$ as ‘failures.’ So, we assign ’successes’ to the data generated from the depleted/null circRNA, denoted by the vectors ${\mathbf{Z}}^*$’s, and the ‘failures’ refer to the observed ${\mathbf{Z}}$. Thus *r*(*z*) is the proportion of successes as a function of *z*.
2. Performs a non-parametric smoothing of the success-failure proportion as a function of *z*.

Further details of this procedure can be found in the Supplementary document.

In the actual implementation, the depleted circRNAs from two of three experimental RNase R-datasets (Hela, Hs68 and Hek293) are used for computing the fdr2d of the remaining dataset (see the Datasets section). For example, the fdr2d values computed for the Hela RNase R-dataset are estimated from the null data collected from the two other RNase R– datasets. Similarly for the Hs68 and Hek293 datasets.

### Circall-simulator

Simulated circRNA data have been used in several studies [1, 9, 29]. Even though the simulation model is made as realistic as possible, simulated data are usually still far from reality. However, as the ground truth of CircRNAs is known and controlled, simulated data are informative for method comparisons, since in these comparisons what matters is the relative rather than the absolute performances.

None of the current simulation tools take the tandem RNAs into account. Thus, we developed Circall-simulator to simulate RNA-seq data of circRNA, tandem RNAs and linear RNAs (Additional file 1: Fig. S4). The circRNAs and tandem RNAs are the main contributors to the proportions of the true positive and false positive in the simulated data, respectively. In brief, the input to the simulator includes a list of circRNAs, tandem RNAs and linear RNAs, and their corresponding expression levels. Then, using the information from gene annotation and the sequences of genes and transcripts, it builds pseudo-sequences of the circRNAs and tandem RNAs (Additional file 1: Fig. S5). Finally, Circall-simulator generates RNA-seq reads for these input transcripts using the RNA simulator Polyester [34]. The tool is implemented as an R package and publicly available for use in Circall’s web page. Further details of Circall-simulator are given in the Supplementary document.

### Datasets

We evaluate the performance of Circall in two simulated datasets and three experimental datasets of human cell-lines including Hela [29], Hs68 [25] and Hek293 [30].

#### Simulated datasets

We collect the outputs of the five methods from the Hs68 dataset (see Results section) for simulation. Specifically, we select 6,256 non-depleted exonic circRNAs found in the circBase [35] detected by either methods as true circRNAs. We also assign 1,008 circRNAs detected in the RNase R- sample but not found in the RNase R+ sample by either methods as tandem RNAs. We run Salmon [36] to get the expression of linear transcripts. Next, the expression (in Fragments Per Kilobase of transcript per Million, FPKM) of circRNAs and tandem RNAs are sampled from the expression distribution of the linear transcripts. To avoid outliers, the range of the distribution is limited from 0.2 to the 99th-percentile of the observed expression distribution. Finally, we generate the paired-end RNA-seq reads of these transcripts using Polyester under these specific settings: sequencing error rate 0.005, read length 100bp, fragment length distribution with mean 250 and standard deviation 25.

We further generate another simulated dataset using third-party simulators following the description in the recent comparative study [9]. Particularly, the same set of circRNAs are simulated with CIRI-simulator with read length 100bp. The linear RNAs are simulated by ART simulator with read length of 100bp using the input of RefSeq mRNA sequences downloaded from the UCSC Genome Browse [37]. The commands to run the tools are provided in the Supplementary document.

We combine the RNA-seq to generate three simulated datasets: (1) Mix1 includes only circRNA and linear transcripts, (2) Mix2 includes Mix1 and tandem RNAs, and (3) Mix3 includes circRNA and linear RNAs generated by CIRI-simulator and ART simulator. Mix1 and Mix3 do not contain tandem RNAs, so Mix2 will help evaluate the circRNA detection methods when there are false-positive BSJ reads from tandem RNAs. Details of the simulated datasets are presented in Table 1.

#### Experimental datasets

The Hela dataset [29] includes four ribosomal-RNA depleted samples: two (SRR1636985 and SRR1636986) are RNase R+ and the other two (SRR1637089 and SRR1637090) are RNase R–. Total RNA was isolated using TRIZOL, and ribosomal RNA was removed using a Ribominus kit to exclude a majority of most abundant ribosomal RNA molecules and to improve detection of less abundant transcripts. RNase R+ samples were incubated at 37 °C for 1 h in 16 μl reaction with 10 U/μg RNase R. The libraries were then prepared using the TruSeq protocol following sequencing by Illumina HiSeq 2000 platform with 101bp paired-end reads [29]. In this study, samples from the same library type are merged as input into circRNA detection methods.

The Hs68 dataset comprises one RNase R– sample (SRR444975) and one RNase R+ sample (SRR445016). RNA was isolated with the RNAeasy system. Ribosomal RNA was removed from total RNA using the RiboMinus kit. 14.3 μl treated with 1 μl water or 1 μl RNase R according to its treatment. The reactions proceeded at 40 °C for 1h before undergoing a modified TruSeq library preparation protocol following sequencing by Illumina HiSeq instrument with 100 bp paired-end reads [25].

The Hek293 dataset contains SRR3479243 for RNase R– sample and SRR3479244 for RNase R+ sample. Total RNA was isolated using TRIZOL, ribosomal RNA was removed using a Ribominus kit, and RNase R+ samples were incubated at 37 °C for 1 hour with 10 U/μg RNase R. The libraries were then prepared and sequenced by TruSeq protocol and Illumina HiSeq 2500 platform. This dataset has paired-end reads of length 150 bp [30].

The summary of the experimental datasets is presented in Table 2. For each detection method, the circRNAs detected from the RNase R– samples are considered as discoveries, while the non-depleted circRNAs found from the RNase R+ samples are considered as validation. For convenience, we only use the terms ‘Hela RNase R+’and ‘Hela RNase R–’ in the context of the separation of the library types in the Hela dataset, otherwise ‘Hela’ refers to the discovery ‘Hela RNase R–’ samples. We apply the similar rule to Hs68 and Hek293 datasets.

### Performance evaluation and competing tools

We compare Circall against the current leading methods, including CIRI2 [19], CIRCexplorer [26, 31], MapSplice [27] and find_circ [15]. The first three methods are the top-performing methods in recent comparative studies [8, 9], whereas find_circ is a widely used circRNA detection tool [10].

Briefly, CIRI2 detects circRNAs based on the paired chiastic clipping (PCC) signals from the mapping information of the reads by BWA-MEM [38]. The algorithm utilises the maximum likelihood method based on multiple-seed matching to identify the BSJ reads, then applies systematic filtering to reduce FPs [19, 29]. CIRCexplorer parses the spliced alignment output of the reads mapped to the genome, using TopHat [39] or STAR aligners [11], for detection of the BSJ reads. Those reads are the ones that are split and mapped to the same chromosome but in reverse order. The canonical splice-site condition is also taken into account. MapSplice identifies multiple types of splice junction events [27]. It segments reads into multiple anchors to detect canonical and non-canonical junctions by employing Bowtie1 aligner [12]. The tool find_circ uses Bowtie2 [40] for read mapping to the reference genome. It first collects unmapped reads and extracts 20-mers from both ends for the second alignment to find the BSJ reads. Then, it extends the anchors’ alignment in the third mapping. Finally, it applies a series of filtering steps to select reliable circRNA candidates [15].

CIRCexplorer version 1.1.10 is applied using STAR aligner as suggested in a recent comparative study [9]. CIRI2 version 2.0.6, MapSplice version 2.2.1, and find_circ version 1.2 are applied with their default settings. The reference genome, transcriptome and genome annotation of hg19 Homo sapiens reference were downloaded from the Ensembl websites (version GRCh37.75). For CIRCexplorer, since it is not able to run with Ensembl annotation, the corresponding annotation version built from the UCSC data sources. The details of the command lines used to run these methods in this study are presented in the supplementary document.

Evaluation of circRNA dectection methods in the experimental datasets is done following a recent comparative study [9]. The comparison is based on the circRNAs discovered from the RNase R– samples. First, the expressions of circRNAs in both the RNase R– samples and the corresponding RNase R+ samples are normalised to their library size. Then CircRNAs of a RNase R– sample are classified as true positive (non-depleted) if their expressions do not fall in the corresponding RNase R+ sample. Finally, we rank the discovered circRNAs of each method and compare the true discovery curves. For Circall, we rank circRNAs by their fdr2d value. The other methods do not score the detected circRNAs, but most recent comparative studies use the circRNA expression to rank them [8–10]. We have followed the same method here. Furthermore, to investigate the efficiency of fdr2d, we also rank the circRNAs of Circall based on the expression value alone; the result is called Circall_Count. Finally, we also compare the number of enriched circRNAs among the top-100 candidates. CircRNAs that are least 5-fold enriched in the RNase R+ sample are classified as significantly enriched.

For the simulated data (Table 3), the methods are compared in terms of sensitivity (recall), precision, and F1 score. Sensitivity is the ratio between the number of the discovered true positives and the total number of true circRNAs. Precision is the ratio between the number of the discovered true positives and the total circular candidates discovered by the cirRNA detection methods. F1 score is defined as

4 $$\begin{aligned} \text{F1} = 2\times \frac{\text{Precision} \times \text{Sensitivity}}{\text{Precision} + \text{Sensitivity}}. \end{aligned}$$

F1 is a balanced metric that takes both precision and sensitivity into account, so it is often more appropriate for overall comparisons.

## Results

### Simulation study

The summary results of Circall, CIRI2, Mapsplice and CIRCexplorer for simulated datasets are reported in Table 3. (Despite our effort, the tool find_circ failed to run on the fastq data produced by Polyester.) In general, all methods achieve comparable precision. For the Mix1 dataset, they reach more than 99% precision. Similarly, in the Mix3 dataset generated by CIRI-simulator, these methods still perform well with greater than 97% precision. For the Mix2 dataset, which contains tandem RNAs, the precision is reduced in all methods to 86%. The number of FPs increases substantially between the Mix1 and Mix2 datasets, so all methods are negatively affected by the tandem RNAs.

However, the sensitivity is much varied. For the Mix1 dataset, Circall, CIRI2 and CIRCexplorer are comparable top performers with more than 89% sensitivity, while Mapsplice has 77.05%. A similar order is found in the Mix3 dataset. Circall and CIRI2 are still the top performing methods with more than 93% sensitivity, followed by CIRCexplorer, find_circ, and Mapsplice with 88.63, 85.92 and 82.27% respectively. Compared to the Mix1 dataset, the sensitivity of the methods in the Mix2 dataset does not change for Circall and CIRI2, but is slightly different for Mapsplice and CIRCexplorer. F1 score is usually a better metric to compare overall performance. The results from Table 3 show that Circall and CIRI2 are the top performers with comparable F1 scores across the three datasets, i.e., 0.95, 0.88 and 0.96 respectively. CIRCexplorer achieves the same F1 score to the two methods in the Mix2 dataset but lower performances in the Mix1 and Mix3 datasets with 0.94 and 0.92, respectively. find_circ obtained 0.93 in the Mix3 dataset and Mapsplice reports the lowest scores of 0.87, 0.88 and 0.90.

**Fig. 3:**
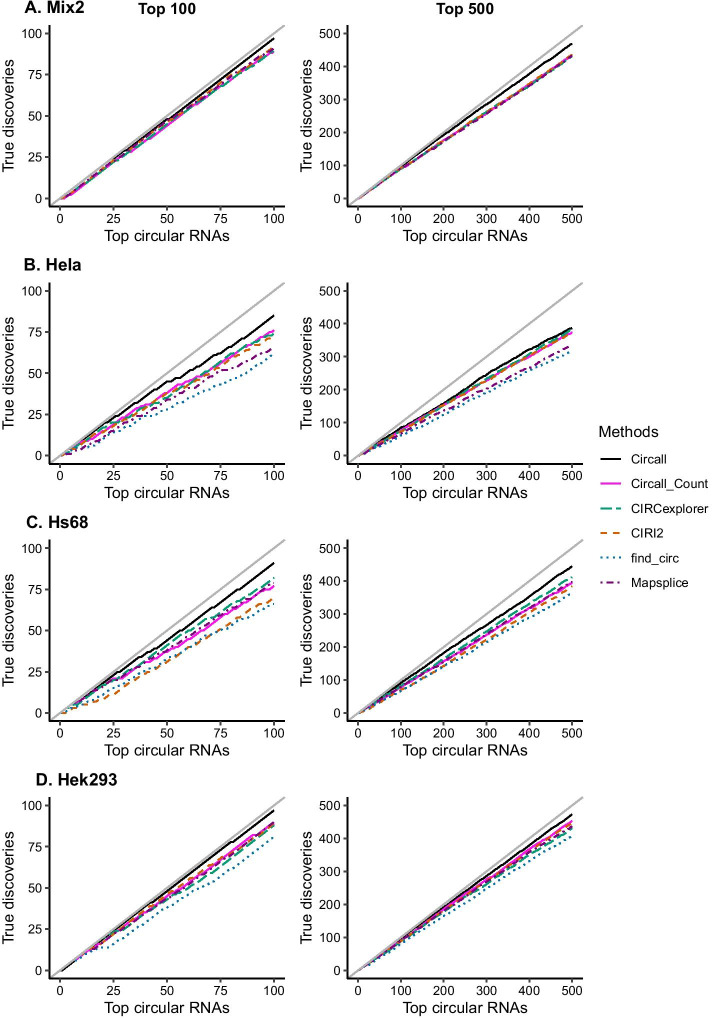
Comparison of the circRNA detection tools in the Mix2 and experimental datasets at the top 100 and 500 circRNAs: (**A**) the Mix2 dataset, (**B**) the Hela dataset, (**C**) the Hs68 dataset, and (**D**) the Hek293 dataset. The x-axis indicates the number of top-ranked detected circRNAs, and the y-axis the number of true positive circRNAs among the top circRNAs. Each curve represents a method. The solid gray line is the diagonal line, which represents 100% true discovery rate. For Circall, circRNAs are ranked by their fdr2d, while for Circall_Count, and the other methods the candidates are ranked by their BSJ supporting-read counts

Figure 3A presents the true discovery curves of Circall as compared to the other methods in the Mix2 dataset. The x-axis presents the number of top circRNAs, while the y-axis presents the corresponding number of true discoveries. Results obtained with the method closest to the diagonal line has the best performance. The plots show the Circall outperforms the other methods in in top 100 and top 500 candidates. Similar results are found then extending to top 1000 and all circRNAs, Additional file 1: Fig. S6.

### Experimental data analyses

In general, Circall performs well against the other methods in all three datasets. Details of the performance in the top 100 and 500 circRNA candidates are reported in Table 4. The plots and results for all detected circRNAs are given in Additional file 1: Fig. S6 and Additional file 1: Table S1 of the Supplementary document.

For the Hela dataset, Circall, CIRCexplorer and CIRI2 are the top performers. For the top 100 candidates, Circall achieves the highest true positive rate with 85% non-depleted circRNAs, followed by CIRCexplorer, CIRI2, Mapsplice and find_circ with 74%, 73%, 66% and 61%, respectively. The curve of Circall is above the others, up to the top 500 candidates, though the distance to the runner-up CIRCexplorer gradually decreases, Fig. 3B. Comparing the discovery of enriched circRNAs among the top 100 candidates, CIRCexplorer and Circall discover 16 and 11 respectively, while the other methods report less than 10 enriched circRNAs (Table 4).

For the Hs68 dataset, the true discovery curve of Circall is close to the diagonal line and remains separated from CIRCexplorer and Mapsclice for up to the top 500 candidates; see Fig. 3C. From Table 4, for the top 100 candidates, Circall has the highest true positive rate with 91 non-depleted circRNAs, followed by CIRCexplorer, Mapsplice, CIRI2 and find_circ with 82, 79, 70 and 66, respectively. Unlike the results from the Hela dataset, in this dataset, CIRI2 is generally worse than Mapsplice. For discovering enriched circRNAs, there is a larger gap between Circall, with 78 enriched circRNAs, and Mapslice and CIRCexplorer, with 62 candidates.

All methods achieve good performance in the Hek293 dataset; see Table 4. For the top 500 candidates, Circall achieves around a 95% true discovery rate, and its curve is consistently above the other methods; see Fig. 3D. Circall detects the highest number of enriched RNAs with 54 circRNAs among the top 100 candidates, significantly higher than CIRCexplorer (36) or find_circ (25).

To investigate the efficiency of the fdr2d procedure, we compare the performances of Circall with Circall_Count, which is based on the circRNAs detected by Circall but using only expression value for ranking. The results in Fig. 3 and Table 4 show that Circall_Count always has lower performances compared to the results of Circall across all datasets, but it is generally comparable with other top performing methods.

### Computational time

**Fig. 4:**
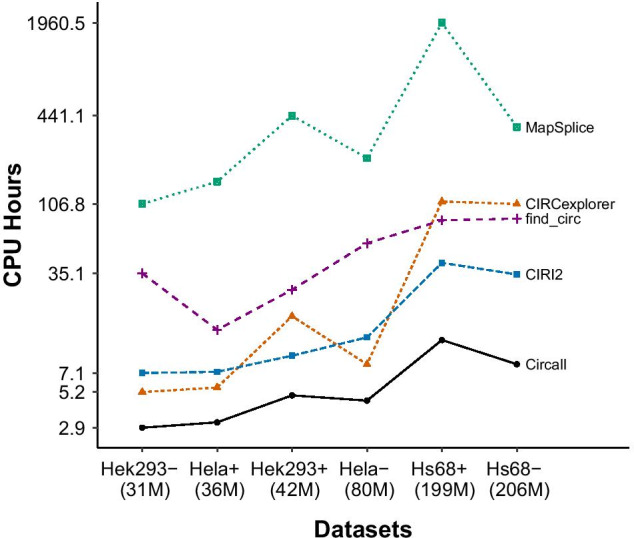
The computational time of circRNA detection methods. Five methods—Circall, CIRCexplorer, CIRI2, find_circ, and MapSplice—are compared by the total CPU time. The datasets in the x-axis are ordered by their library sizes (million reads), and the CPU time on the y-axis is given in log-scale

We measure the total CPU time of all methods for individual datasets, which are reported in Fig. 4 and Additional file 1: Table S2. The time is calculated from the time the methods start running until results are produced. It covers all the steps of a circRNA detection tool including read alignment, filtering, etc. All runs are implemented in the Rackham cluster from the Uppsala Multidisciplinary Center for Advanced Computational Science (UPPMAX - https://www.uppmax.uu.se/) using the computer nodes with Intel Xeon E5 2630 v4 at 2.20 GHz/core under CentOS 7. All tools are run in parallel with 8 cores, each core has 16 GB memory; the reported time is in core-hours.

Figure 4 shows that, as expected, the timing of most methods is linear in library size. Circall is generally faster than the other methods. CIRCexplorer and CIRI2 are comparable depending on the datasets and overall better than find_circ, while the most time-consuming method is Mapsplice. For the smallest dataset, Hek293 RNase R–, Circall (2.9 h) is $\sim$2 times faster than CIRCexplorer (5.2 h) and CIRI2 (7.1 h), and more than one order of magnitude faster than find_circ (35.1 h) and MapSplice (106.8 h). For the large dataset (199M reads), Hs68 RNase R+, Circall requires 12.0 h, while CIRI2, find_circ, CIRCexplorer and MapSplice require 41.4, 82.1, 111.1 and 1960.5 h, respectively. The details of the time consumption are provided in Additional file 1: Table S2.

## Discussion and conclusion

We have developed a novel method, Circall, for fast and accurate detection of circRNAs. We compare the performances of Circall versus current leading methods including CIRI2, CIRCexplorer, MapSplice and find_circ using both simulated and experimental datasets. The results show that Circall achieves high sensitivity and precision in simulation studies, and performs well against existing circRNA detection methods in the analyses of experimental datasets. Computationally, as it is based on an ultra-fast quasi-mapping algorithm, Circall is substantially faster than other methods, particularly for large datasets.

The main advantage of Circall is the scoring system using fdr2d based on the read-count and circRNA-length statistics. The read count is a conventional factor that has been used as evidence by most circRNA detection tools. It is also used to rank the circRNAs in most methods when exporting results. CircRNA length has also been a key feature in previous studies. Most circRNAs in human cells are a few hundred nucleotides [16, 18, 24–26]. Furthermore, circRNAs detected with short distances between back splice sites tended to be FPs, specifically, almost no circRNAs with splice site distances less than 200 bases was characterised as *bona fide* circles [8]. Our study demonstrates that combining information on the circRNA length and supporting reads can help to improve circRNA detection accuracy.

**Table 1:** Details of the simulated datasets

Dataset	Read length (bp)	Library size	Number of BSJ
CircRNA	100	997,486	6256
CircRNA - CIRI-simulator	100	1,072,012	6256
Tandem RNA	100	1,623,654	1008
Linear RNA	100	112,614,394	0
Linear RNA - ART simulator	100	170,541,399	0
Mix1	100	113,611,880	6256
Mix2	100	115,235,534	6256
Mix3	100	171,613,411	6256

Mix1 includes circRNAs and linear RNAs, Mix2 is the extension of Mix1 with tandem RNAs, and Mix3 consists of the same set of circRNAs from Mix1 but generated by CIRI-simulator, and linear RNAs produced by ART simulator

**Table 2:** Details of the experimental datasets

Dataset	Read length (bp)	Library size	SRA accession number
Hela RNase R–	101	80,618,760	SRR1637089, SRR1637090
Hela RNase R+	101	36,815,458	SRR1636985, SRR1636986 ara>
Hs68 RNase R–	100	206,362,733	SRR444975
Hs68 RNase R+	100	199,922,486	SRR445016
Hek293 RNase R–	150	31,059,167	SRR3479243
Hek293 RNase R+	150	42,307,449	SRR3479244

The Hela samples are combined for the circRNA detection

**Table 3:** Results of Circall, CIRI2, Mapsplice, CIRCexplorer and find_circ in the simulated datasets

Dataset	Method	# detected circRNA	#False positive	#true circRNA	Sensitivity (%)	Precision (%)	F1
Mix1	Circall	5644	10	5634	90.06	99.82	**0.95**
	CIRI2	5661	15	5646	90.25	99.74	**0.95**
	Mapsplice	4822	2	4820	77.05	99.96	0.87
	CIRCexplorer	5631	25	5606	89.61	99.56	0.94
Mix2	Circall	6503	869	5634	90.06	86.64	**0.88**
	CIRI2	6531	885	5646	90.25	86.45	**0.88**
	Mapsplice	5603	782	4821	77.06	86.04	0.81
	CIRCexplorer	6522	917	5605	89.59	85.94	0.88
Mix3	Circall	5940	79	5861	93.69	98.67	**0.96**
	CIRI2	6062	163	5912	94.29	97.31	**0.96**
	Mapsplice	5148	1	5130	82.27	99.98	0.90
	CIRCexplorer	5414	39	5372	85.92	99.28	0.92
	find circ	5729	184	5545	88.63	96.79	0.93

The values with bold text in the F1 column indicate the top-performing methods in the dataset

**Table 4:** Results of Circall, Circall_Count, CIRI2, Mapsplice, CIRCexplorer and find_circ in the experimental datasets

Dataset	Method	Enrichment in top 100	Top 100		Top 500	
			Non-dep	Percent (%)	Non-dep	Percent (%)
Hela	Circall	11	**85**	**85.0**	**387**	**77.4**
	Circall_Count	6	76	76.0	373	74.6
	CIRI2	7	73	73.0	375	75.0
	find_circ	4	61	61.0	317	63.4
	Mapsplice	1	66	66.0	337	67.4
	CIRCexplorer	**16**	74	74.0	383	76.6
Hs68	Circall	**78**	**91**	**91.0**	**445**	**89.0**
	Circall_Count	61	77	77.0	395	79.0
	CIRI2	55	70	70.0	384	76.8
	find_circ	52	66	66.0	365	73.0
	Mapsplice	62	79	79.0	398	79.6
	CIRCexplorer	62	82	82.00	412	82.4
Hek293	Circall	**59**	**97**	**97.0**	**473**	**94.6**
	Circall_Count	31	89	89.0	453	90.6
	CIRI2	34	89	89.0	447	89.4
	find_circ	25	81	81.0	407	81.4
	Mapsplice ara>	26	90	90.0	437	87.4
	CIRCexplorer	36	88	88.0	430	86.0

Column “Non-dep” indicates the number of non-depleted circRNAs and column “Percent” shows the percentage of the non-depleted circRNAs in the top circRNAs. The values with bold text indicate the top-performing methods in the dataset

Most circRNA detection methods dealing with FP circRNAs based on classifying BSJ supporting-reads to true circRNAs or tandem RNAs. As illustrated in the lower dashed box Fig. 2, if the mate of a BSJ supporting-reads from a tandem RNA belongs to the region of the true circRNA, the mapping information alone cannot tell if the read-pair comes from a true circRNA or from a tandem RNA. It usually happens when either two reads come from a short fragment or the circRNA length is too long. This explains why long circRNAs are hard to predicted from RNA-seq data. The fdr2d cannot resolve this problem caused by the long circRNAs, but it provides a ranking system to prioritize circRNAs based on false discovery rate. It is worth noting that fdr2d does not down-prioritize the long circRNAs in terms of biological aspects, thus if researchers have special interests in the long circRNAs, they could treat them separately. Circall also provides the information of circRNA length in its output. The performances of fdr2d aslo depend on the previously published datasets that we expect they could capture a well representation of data. In this study, only two out of three real datasets are used to build the model for the results of the remaining dataset, thus the model is independent from the testing data. The results show the model work robustly to all datasets in this study.

Circall also has some weaknesses. First, it detects only exonic circRNAs, while neglecting the potential contribution of introns. However, from previous studies [3, 18], most circRNAs are derived from exons, and back-splicing events occur mostly at annotated exon boundaries. Further study to discover non-exonic circRNAs can be considered in future works. Second, Circall builds the BSJ database based on the annotated reference, thus depends on the completion of the annotation. This cannot be avoided for any reference-based approach methods, but we expect the annotation reference would continue to be improved in the future. The rapid development of long-read sequencing technologies is expected to significantly improve the gene annotation [41]. The advancement of the technology recently helped to detect more complex structures of circRNAs [42]. Finally, Circall currently works for paired-end RNA-seq data only, so single-end RNA-seq data generated from old RNA-seq protocols are not allowed. However, paired-end RNA-seq for total RNAs is now more prevalent in analyses of circRNAs, so this is not a serious limitation.

## Supplementary Information

**Additional file 1.** The supplementary document includes the details of the fdr2d method, the Circall simulator, supplementary figures and supplementary tables.

## Data Availability

The source codes and bioinformatics pipeline of Circall are available at https://www.meb.ki.se/sites/biostatwiki/circall and https://github.com/datngu/Circall under a GPL-3 license.The source codes to generate the results of Circall in this study are deposited in the Zenodoo repository at https://doi.org/10.5281/zenodo.4719119.

## Abbreviations

RNA-seq: RNA sequencing
BSJ: Back-splicing junction
FP: False positive
circRNA: Circular RNA
FDR: False discovery rate
fdr2d: Two-dimensional local false discovery rate
