# Supplementary material for: Circall: fast and accurate methodology for discovery of circular RNAs from paired-end RNA-sequencing data
Source: BMC Bioinformatics. 2021 Oct 13;22:495. doi: 10.1186/s12859-021-04418-8 (PMC8513298; doi:10.1186/s12859-021-04418-8)
Supplement: Supplementary file 1 — Additional file 1. The supplementary document includes the details of the fdr2d method, the Circall simulator, supplementary figures and supplementary tables. [file 12859_2021_4418_MOESM1_ESM.pdf]

Supplementary documents

Circall: fast and accurate methodology for discovery of circular RNAs from paired-end RNA-sequencing data

By Dat Thanh Nguyen, et al

1 Details of two-dimensional local false discovery rate estimation

Here we describe in detail the estimation of two-dimensional local false discovery rate (fdr2d) in the context of circRNA detection from paired-end RNA-seq data. For completeness and clarity, some parts from the main text are repeated.

Suppose there are n the non-depleted circRNA candidates detected from the RNA-seq data and z values are the observed statistics of these circRNAs. Let $\mathbf{Z}$ be the vector of n observed z values. Assume that each non-depleted circRNA contains the pair of statistics (z_1, z_2). The estimation of $f_0(z)$ is based on the data of M random samples, each of size n, of the null circRNAs, herein the depleted circRNAs. Denote these samples as $\mathbf{Z}_1^*, \ldots, \mathbf{Z}_M^*$, representing samples of $\mathbf{Z}$ under the null hypothesis of depleted circRNAs. In all experiments of this study we use $M = 100$ samples.

First, the procedure estimates the ratio

$$r(z) \equiv \frac{Mf_0(z)}{f(z) + Mf_0(z)},$$

then computes the fdr2d as

$$\text{fdr2d}(z) = \pi_0 \frac{r(z)}{M\{1 - r(z)\}}, \tag{1}$$

In order to estimate the 2d-estimation of $r(z)$, the procedure

1. considers all the statistics from $\mathbf{Z}_1^*, \ldots, \mathbf{Z}_M^*$ as 'successes' and the observed statistics from $\mathbf{Z}$ as 'failures', so that $r(z)$ is the proportion of successes as a function of z.

2. performs a nonparametric smoothing of the success-failure proportion as a function of z.

Because the full set of null data $\mathbf{Z}_1^*, \ldots, \mathbf{Z}_M^*$ is large, to speed up computations, we pre-bin the data of the two dimensional grids, and the smoothing procedure to estimate the fdr2d is implemented on the grids.

From hereon the theory follows Pawitan (Pawitan, 2001), section 18.10. Let y_{ij} be the number of successes in the (i, j) location of the grid, and N_{ij} the corresponding total number of points that fall in the (i, j) location. By construction, y_{ij} is binomial with size N_{ij} and probability r_{ij}. Probability r_{ij} is the discretized version of $r(z)$ or r for simplicity. Given a smoothing parameter λ, the smoothed estimate of r_{ij} is the minimizer of the penalized log-likelihood

$$\log L(r, \lambda) = -\sum_{ij} \{y_{ij} \log r_{ij} + (N_{ij} - y_{ij}) \log(1 - r_{ij})\} + \lambda \sum_{(i,j) \sim (k,l)} (\eta_{ij} - \eta_{kl})^2$$

where $(i, j) \sim (k, l)$ means that (i, j) and (j, k) are primary neighbors in the 2D grids , and

η_{ij} is a link function $h(r_{ij})$. In practice, we use the identity link function $h(r_{ij}) = r_{i,j}$. The estimate is computed using the iteratively weighted least-squares (IWLS) algorithm, which is a very stable algorithm in this case. Define the following arrays:

$$\begin{aligned} Y &\equiv \text{vec}(y_{ij}) \\ r &\equiv \text{vec}(r_{ij}) \\ \Sigma &\equiv \text{Diag}[N_{ij} r_{ij}(1 - r_{ij})], \end{aligned}$$

and R is the relationship matrix representing the primary neighbors (i.e. North, South, East and West) in the 2D grids. Let k or l be the 1D index of vector Y; the elements of $R^{-1} \equiv \text{Diag}[e_{kl}]$ are given by

$$\begin{aligned} e_{kk} &= \text{number of primary neighbors of } k \\ e_{kl} &= -1 \text{ if } l \text{ is a primary neighbor of } k, \text{and } 0 \text{ otherwise.} \end{aligned}$$

Starting with an initial estimate $r^{(0)}$ needed to compute the variance matrix Σ, the IWLS updating equation is

$$r^{(1)} = (\Sigma^{-1} + \lambda R^{-1})^{-1} \Sigma^{-1} Y.$$

For speed, a fast inversion algorithm based on the Gauss-Seidel algorithm is used. At convergence, the output of the algorithm is a smooth estimate of $r(z)$, evaluated at discrete points (i, j). The fdr2d is then computed using (1), then interpolated at each observed z. The amount of smoothing as a function of λ is assessed by the effective number of degrees of freedom (df), computed using

$$\text{df} = \text{trace}\{(\Sigma^{-1} + \lambda R^{-1})^{-1} \Sigma^{-1}\}.$$

In practice, we use a relatively coarse grid on the order of $15\times$ 15 points, and λ is chosen so that df is approximately 70-80% of the number of grid points.

2 Circall simulator

The tool is implemented as an R package that requires several inputs including circRNA candidate list and the read counts of the circRNAs. Genome annotation, including genome reference, transcriptome reference, and gene annotation file with Sqlite formatted, are also needed to obtain gene models and exon sequences. Because alternative splicing events are also prevalent in CircRNA formation (Gao et al., 2016; Zhang et al., 2016). In cases of more than one linear transcript supporting the BSJ of a circRNA, one linear transcript is randomly selected and its exons are used for the simulation. The simulation package starts by dividing the circRNA list into two lists that used for circRNA and tandem RNA simulation by the user's pre-set tandem rate. The lists of candidates are then followed two sub-pipelines in the package to simulate circRNA and tandem RNA seq data (Figure S.4).

In each sub-pipeline, there are two main steps in the simulation process (i) getting transcript sequences and (ii) simulating synthetic read pairs by Polyester (Frazee et al., 2015). Polyester is a tool that is usually used to generate wild type linear RNAs. By utilizing this package, Circall simulator provides users numerous choices for simulation experiments such as sequencing error rate, read length, and fragment length distribution.

Due to the loop structure of RNA circles, a procedure is employed to mimic the breaking process of circRNAs into linear forms. The collected exon sequences are first concatenated together as linear forms and then randomly cleaved into two sequences. These sequences are then concatenated again in the reverse order to obtain circRNA sequences (Figure S.5 A), and finally used to simulate a synthetic read pair by Polyester package. This procedure is applied every read pair of the target circRNA.

For tandem RNAs, the process is simpler. Based on BSJ information, the collected exon sequences are classified as two types. The first one is the circRNA region, and the other is the non-circRNA region. Transcript sequences are obtained by duplicating the exons in the circRNA region, and then all parts are concatenated together (Figure S.5 B). Because tandem RNA is already in linear forms, synthetic RNA seq reads can directly be simulated by Polyester.

Tables

Table S. 1: Results of Circall, CIRI2, Mapsplice, CIRCexplorer and find_circ in the real datasets at the top 1000 and all circRNAs.

Dataset	Method	Top 1000		All candidates		
		Non-dep	Percent (%)	Non-dep	Detected	Percent (%)
Hela	Circall	751	75.10	5429	13384	40.56
	CIRI2	744	74.40	3199	5844	54.74
	find_circ	609	60.90	2318	6722	34.48
	Mapsplice	667	66.70	1754	3256	53.87
	CIRCexplorer	685	68.50	3528	8261	42.71
Hs68	Circall	873	87.30	6348	10223	62.10
	CIRI2	773	77.30	3300	4569	72.23
	find_circ	735	73.50	2727	4880	55.88
	Mapsplice	789	78.90	1817	2374	76.54
	CIRCexplorer	797	79.70	4830	8009	60.31
Hek293	Circall	865	86.50	3765	7623	49.39
	CIRI2	868	86.80	3002	4216	71.20
	find_circ	752	75.20	1983	3607	54.98
	Mapsplice	809	80.90	1459	2012	72.51
	CIRCexplorer	797	79.70	3439	7216	47.66

Non-dep: number of non-depleted circRNA

Table S. 2: CPU time (hours) of the circRNA detection methods for the analyses of 6 experimental datasets.

Method	Hek293	Hela	Hs68	Library type
Circall	2.9	4.5	8.1	RNase −
CIRI2	7.1	12.5	34.4	
CIRCexplorer	5.2	8.1	106.7	
find_circ	35.1	56.8	84.4	
MapSplice	106.8	223.1	416.0	
Circall	4.9	3.2	12.0	RNase +
CIRI2	8.1	7.2	41.5	
CIRCexplorer	17.6	5.6	111.1	
find_circ	29.6	14.1	82.1	
MapSplice	441.1	152.9	1960.5	

Figures

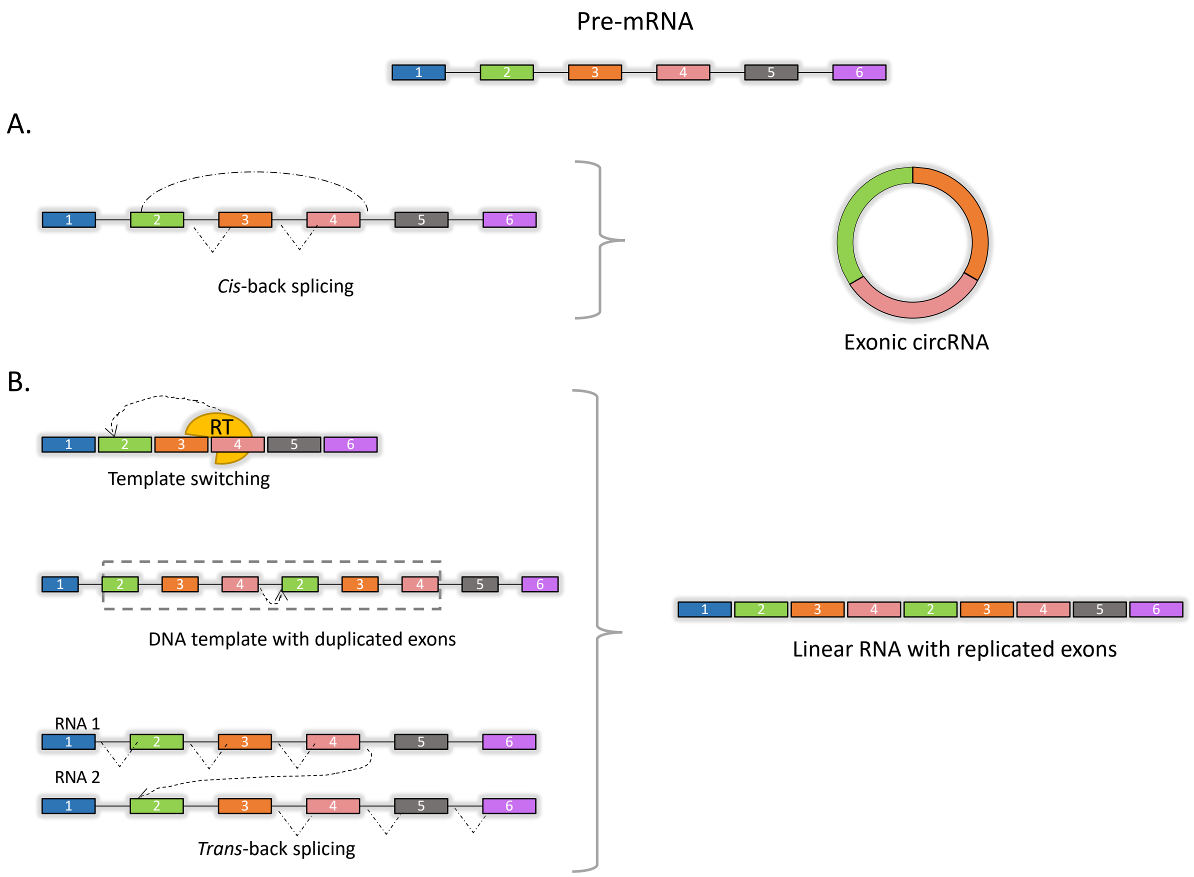

Figure S. 1: Source of true positive and false positive back splicing junctions. A gene model has 6 exons (rectangles) and 5 introns (thin lines). pre-mRNA is showed as series of exons with intron interval while mature RNA is a continuous series of adjacent rectangles of exons. (A) Exonic circRNAs are formed by cis-back splicing in which exons (exon 4 and exon 2) from the same RNA molecule are spliced together to form a circle. (B) Reverse transcriptase (RT) template switching, tandem duplications in the DNA template, trans-back splicing are resulting tandem RNA structure that the exons of the circRNA are duplicated. This also generate a junction region between exon 4 and exon 2

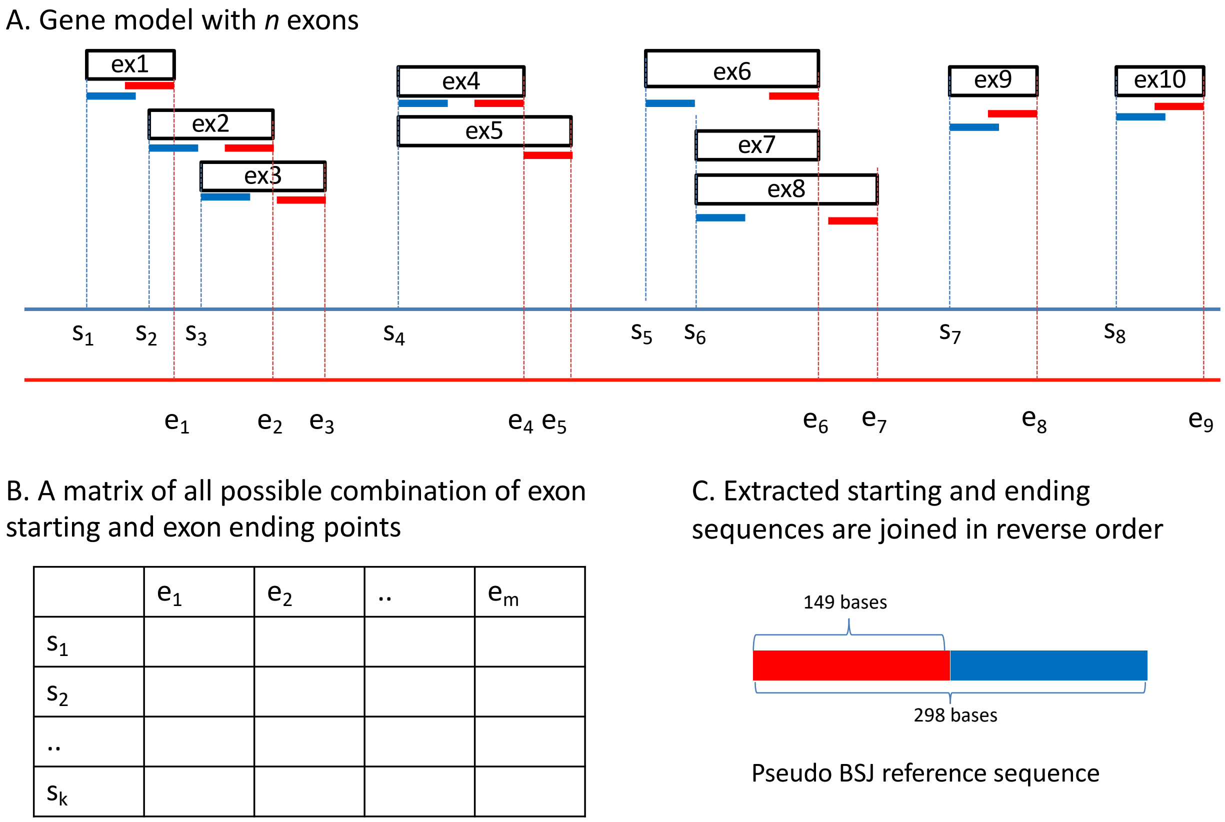

Figure S. 2: Illustration for the process of generating pseudo BSJ reference sequences. (A) A gene model with n=10 exons in rectangles. The solid horizontal lines are the reference genome and the dash vertical lines indicate the unique ending and starting position in the references. The red and blue indicate starting and ending extracted sequences. (B) A matrix of all possible combinations of exon starting and exon ending points. (C) Extracted starting and ending sequences are joined in reverse order in order to generate BSJ reference sequences.

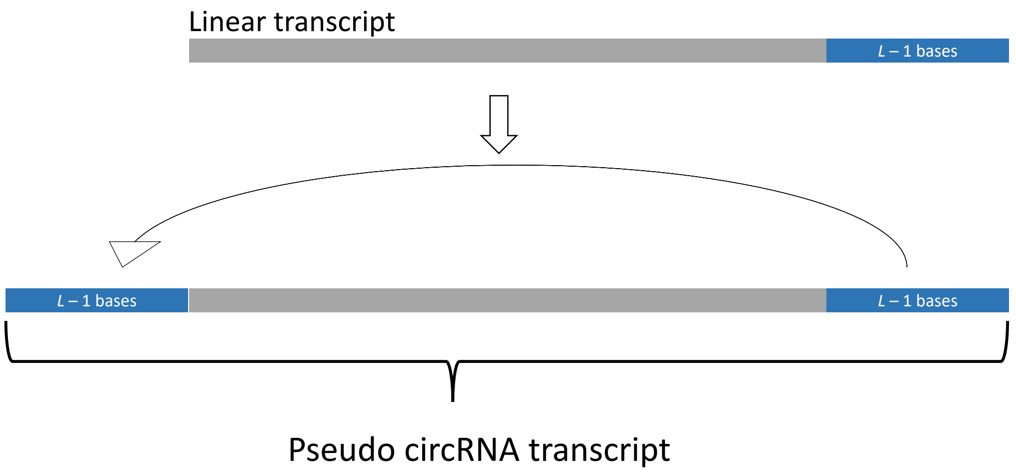

Figure S. 3: Illustration for the process of generating pseudo circRNA transcripts, $L-1$ last bases the sequence are duplicated and joined to the beginning of the sequences, where L is the read length of the RNA-seq data.

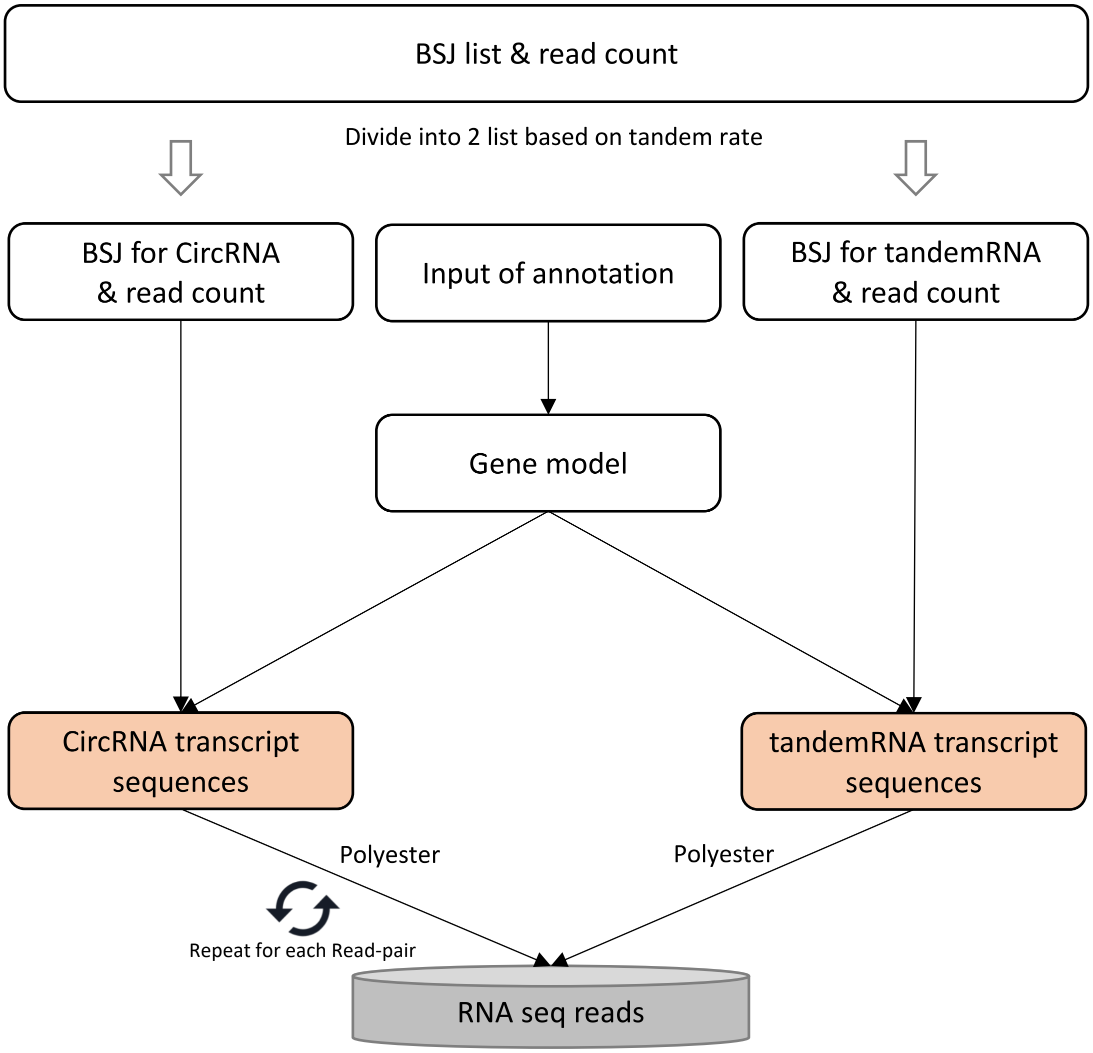

Figure S. 4: Workflow for simulation circRNA and tandem RNA seq data. The input circRNA candidate list is divided into two sub-lists for circRNA and tandem RNA simulation by the user's pre-set tandem rate. They are followed their own sub-pipeline to obtain transcript sequences and then end up by simulating RNA seq reads by Polyester package.

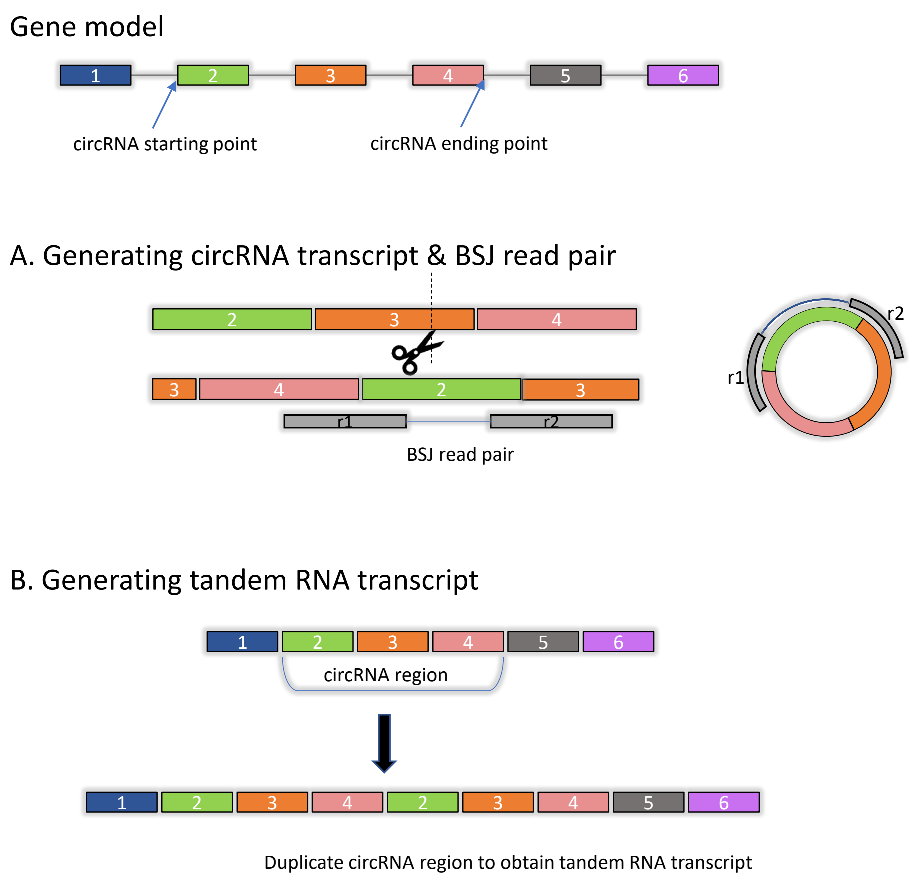

Figure S. 5: Illustration for generating pseudo-sequences of circRNA and tandem RNA. A gene model has 6 exons presented in rectangles, 5 introns in thin lines. The target circRNA includes exons 2, 3 and 4. In panel A: generating a read pair for circRNA. First we linearly combine exons together, randomly cleave into two sequences, the concatenate again in reverse direction to get the circRNA sequence. Panel B: Generating tandem RNA transcripts by simply duplicating the exons in the circRNA region.

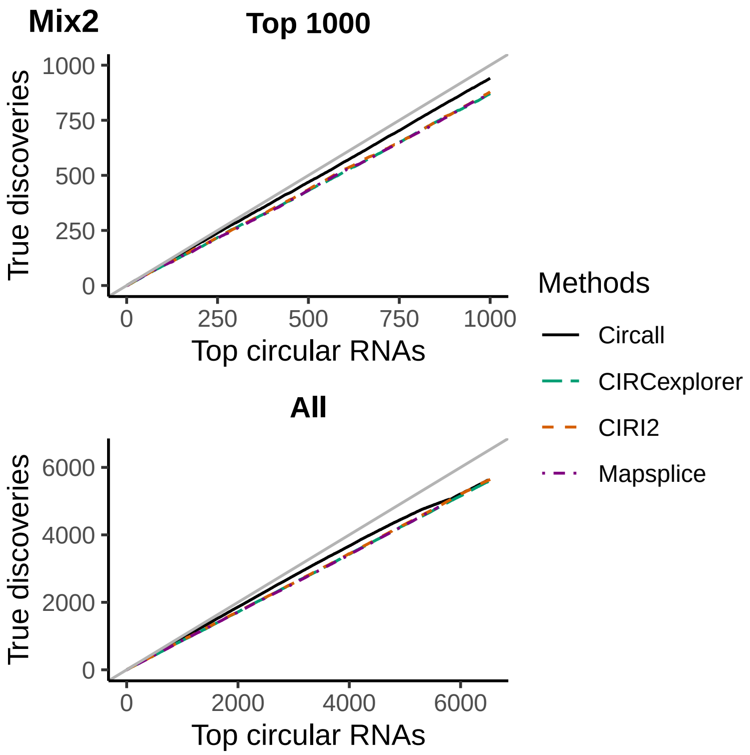

Figure S. 6: Comparison of the circRNA detection tools in the Mix2 datasets at the top 1000 and all circRNAs. The x-axis indicates the indexes of top-ranked circRNAs. The y-axis presents the number of true positive circRNAs in the top circRNAs. The curves represents the true discoveries of the methods. The solid gray line is the diagonal line which represents the perfect true discovery rate (100%). For Circall, circRNAs are ranked by their fdr2d while circRNA candidates of the other methods are ranked by their supporting BSJ spanning read counts.

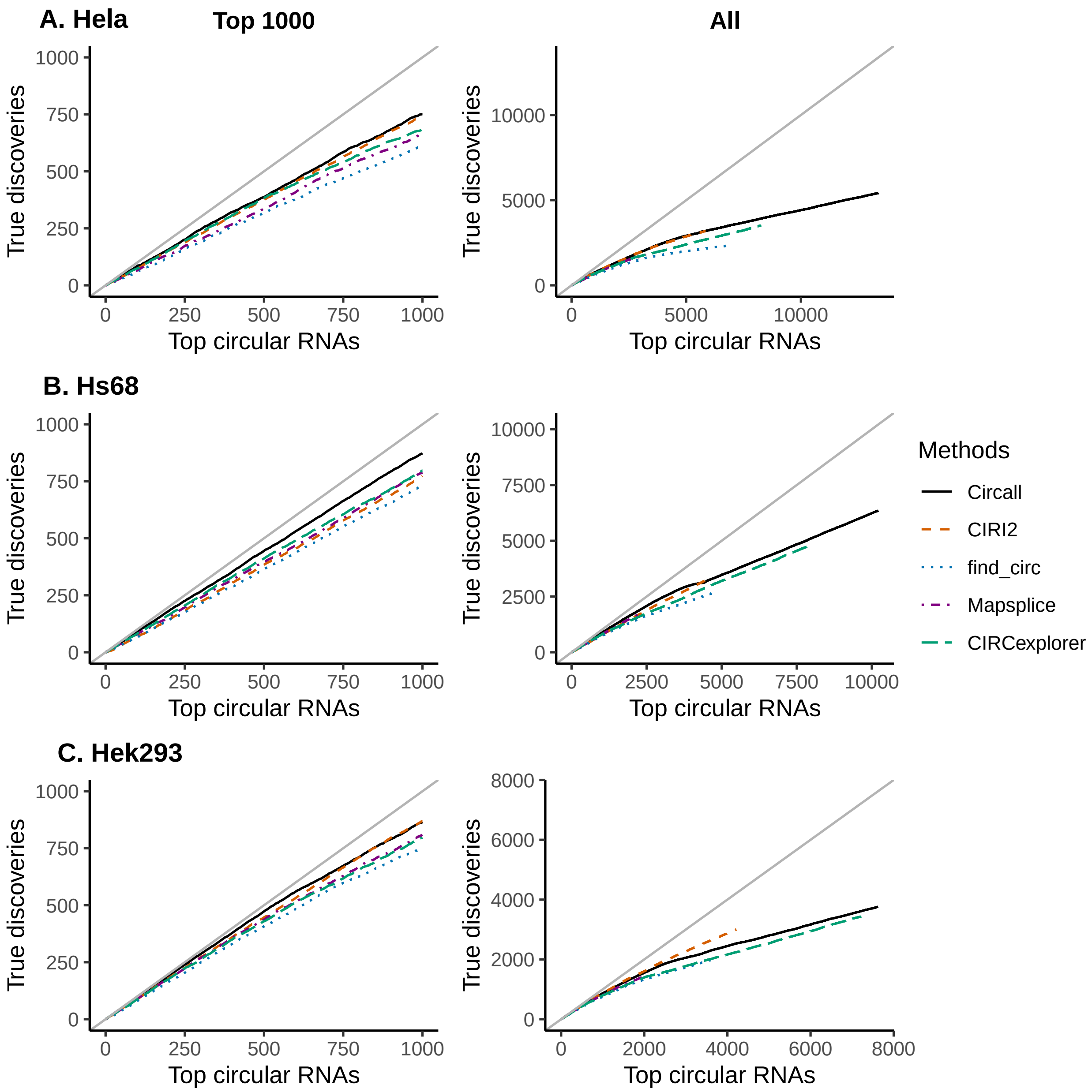

Figure S. 7: Comparison of the circRNA detection tools in the real datasets at the top 1000 and all circRNAs: **(A)** the Hela dataset, **(B)** the Hs68 dataset, and **(C)** the Hek293 dataset. The x-axis indicates the indexes of top-ranked circRNAs. The y-axis presents the number of true positive circRNAs in the top circRNAs. The curves represents the true discoveries of the methods. The solid gray line is the diagonal line which represents the perfect true discovery rate (100%). For Circall, circRNAs are ranked by their fdr2d while circRNA candidates of the other methods are ranked by their supporting BSJ spanning read counts.

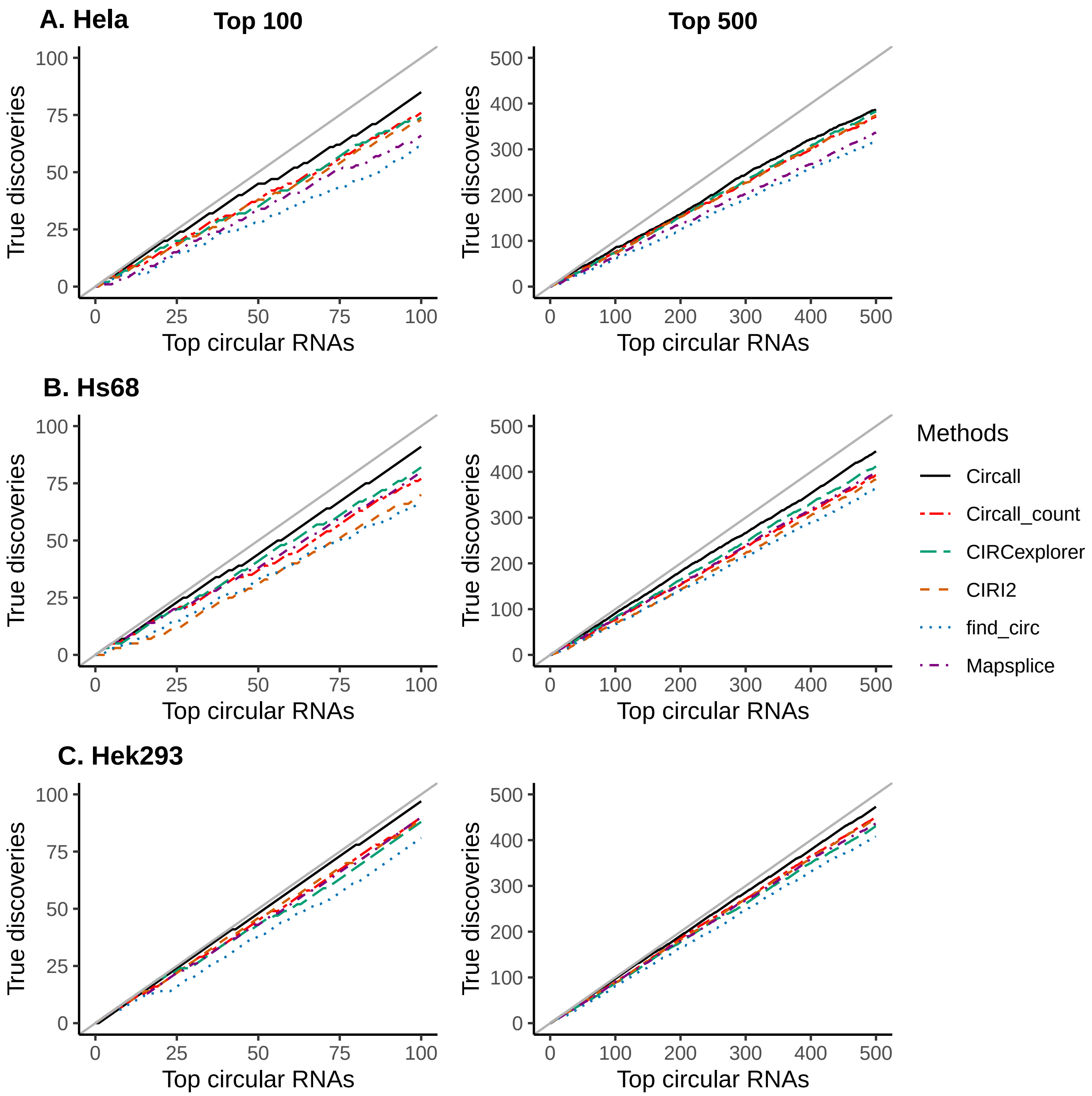

Figure S. 8: Comparison of the circRNA detection tools in the real datasets at the top 100 and 500 circRNAs: **(A)** the Hela dataset, **(B)** the Hs68 dataset, and **(C)** the Hek293 dataset. The x-axis indicates the indexes of top-ranked circRNAs. The y-axis presents the number of true positive circRNAs in the top circRNAs. The curves represents the true discoveries of the methods. The solid gray line is the diagonal line which represents the perfect true discovery rate (100%). For Circall, circRNAs are ranked by their fdr2d while circRNA candidates of Circall_count and the other methods are ranked by their supporting BSJ spanning read counts.

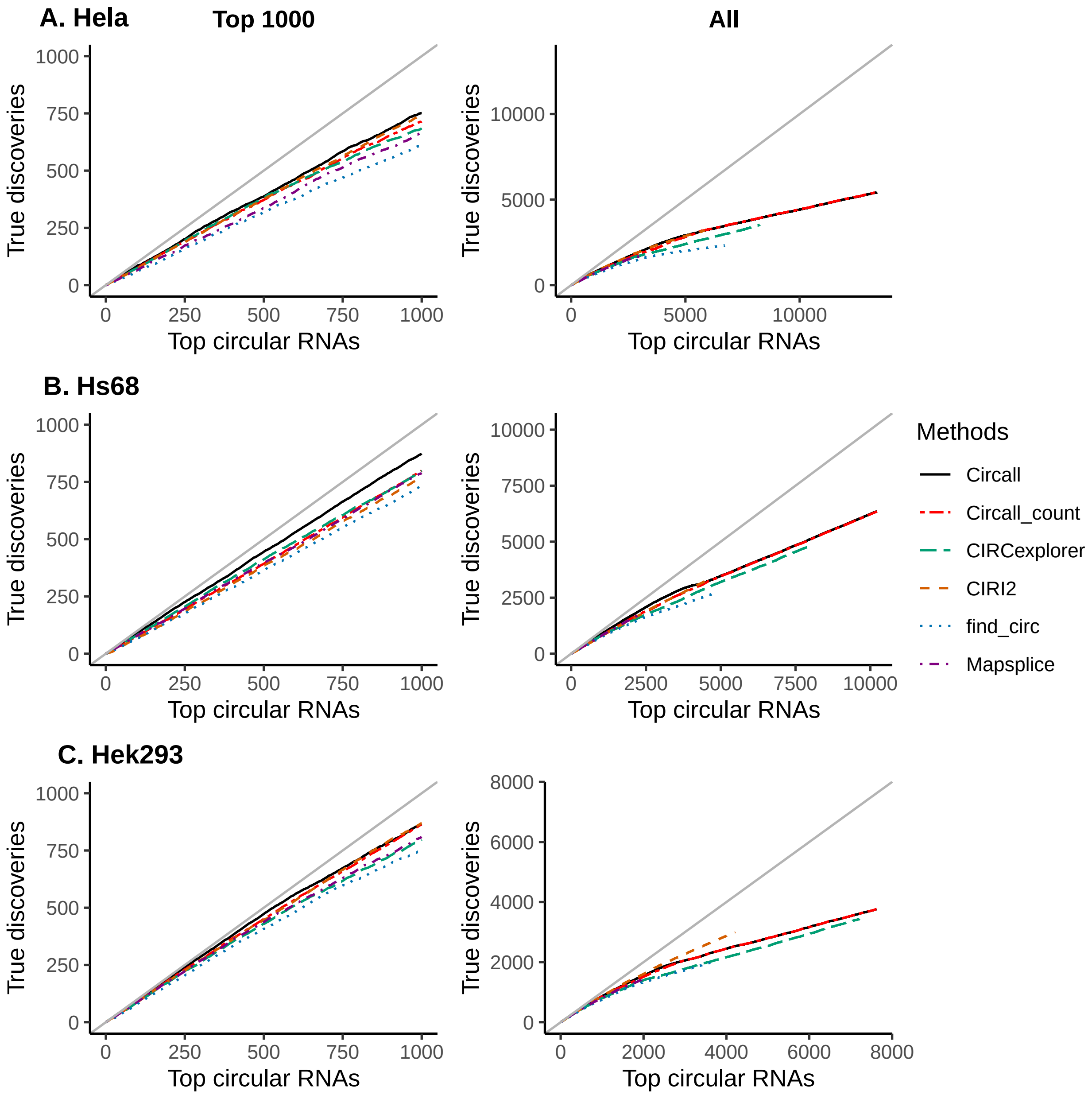

Figure S. 9: Comparison of the circRNA detection tools in the real datasets at the top 1000 and all circRNAs: **(A)** the Hela dataset, **(B)** the Hs68 dataset, and **(C)** the Hek293 dataset. The x-axis indicates the indexes of top-ranked circRNAs. The y-axis presents the number of true positive circRNAs in the top circRNAs. The curves represents the true discoveries of the methods. The solid gray line is the diagonal line which represents the perfect true discovery rate (100%). For Circall, circRNAs are ranked by their fdr2d while circRNA candidates of Circall_count and the other methods are ranked by their supporting BSJ spanning read counts.

References

Frazee, A. C., A. E. Jaffe, B. Langmead, and J. T. Leek, 2015: Polyester: simulating rna-seq datasets with differential transcript expression. *Bioinformatics*, **31 (17)**, 2778–2784.

Gao, Y., J. Wang, Y. Zheng, J. Zhang, S. Chen, and F. Zhao, 2016: Comprehensive identification of internal structure and alternative splicing events in circular rnas. *Nature communications*, **7**, 12 060.

Pawitan, Y., 2001: *In all likelihood: statistical modelling and inference using likelihood.* Oxford University Press.

Zhang, X.-O., R. Dong, Y. Zhang, J.-L. Zhang, Z. Luo, J. Zhang, L.-L. Chen, and L. Yang, 2016: Diverse alternative back-splicing and alternative splicing landscape of circular rnas. *Genome research*, **26 (9)**, 1277–1287.
